# Antiretroviral therapy outcomes among HIV infected clients in Gweru City, Zimbabwe 2006 - 2011: a cohort analysis

**DOI:** 10.11604/pamj.supp.2017.27.1.12562

**Published:** 2017-05-28

**Authors:** Gerald Shambira, Notion Tafara Gombe, Casey Daniel Hall, Meeyoung Mattie Park, Joseph Asamoah Frimpong

**Affiliations:** 1 Zimbabwe Field Epidemiology Training Program, Zimbabwe; 2 Emory University, Atlanta, USA; 3 Liberia Field Epidemiology Training Program, Liberia

**Keywords:** Public Health, epidemiology, antiretroviral therapy, HIV, Zimbabwe, people

## Abstract

The government of Zimbabwe began providing antiretroviral therapy (ART) to People Living with HIV/AIDS (PLHIV) in public institutions in 2004. In Midlands province two clinics constituted the most active HIV care service points, with patients being followed up through a comprehensive patient monitoring and tracking system which captured specific patient variables and outcomes over time. The data from 2006 to 2011 were subjected to analysis to answer specific research questions and this case study is based on that analysis. The goal of this case study is to build participants' capacity to undertake secondary data analysis and interpretation using a dataset for HIV antiretroviral therapy in Zimbabwe and to draw conclusions which inform recommendations. Case studies in applied epidemiology allow students to practice applying epidemiologic skills in the classroom to address real-world public health problems. Case studies as a vital component of an applied epidemiology curriculum are instrumental in reinforcing principles and skills covered in lectures or in background reading. The target audience includes Field Epidemiology and Laboratory Training Programs (FELTPs), university students, district health executives, and health information officers.

## How to use this case study

**General instructions:** case studies in applied epidemiology allow students to practice applying epidemiologic skills in the classroom to address real-world public health problems. Case studies are used as a vital component of an applied epidemiology curriculum, rather than as stand-alone tools. They are well suited to reinforcing principles and skills already covered in a lecture or in background reading. Ideally, 1-2 instructors facilitate the case study for 8 to 20 students in a classroom or conference room. Traditionally, the instructor directs a participant to read aloud a paragraph or two, going around the room and giving each participant a chance to read. When the participant reads a question, the instructor directs all participants to perform calculations, construct graphs, or engage in a discussion of the answer. Sometimes, the instructor can split the class to play different roles or take different sides in answering the question. As a result, participants learn from each other, not just from the instructors.

**Audience:** residents in Field Epidemiology Training Programs (FETPs), Field Epidemiology and Laboratory Training Programs (FELTPs), university students, district health executives, health information officers from the public health sector, and other partner organizations at national and regional level. Participants will have basic graduate qualification in health-related field, e.g. medical degree, nursing, environmental health, social science.

**Prerequisites:** before using this case study, case study participants should have received lectures or other instruction in basic epidemiology, statistics, and secondary data analysis.

**Materials needed:** white board or flip chart and markers, graph paper, computers with MS Excel (optional)

**Level of training and associated public health activity:** advanced – epidemiology and public health research

**Time required:** approximately 3 hours

**Language:** English

## Case study material

- Download the case study student guide (PDF - 2.28 MB)
- Request the case study facilitator guide
